# Biochemical responses of hairgrass (*Deschampsia caespitosa*) to hydrological change

**DOI:** 10.3389/fpls.2022.987845

**Published:** 2022-09-26

**Authors:** Qiaoyu Luo, Yonggui Ma, Zhi Chen, Huichun Xie, Yanlong Wang, Lianyu Zhou, Yushou Ma

**Affiliations:** ^1^School of Life Sciences, Qinghai Normal University, Xining, China; ^2^Qinghai Provincial Key Laboratory of Medicinal Plant and Animal Resources of Qinghai-Tibet Plateau, Qinghai Normal University, Xining, China; ^3^Academy of Plateau Science and Sustainability, Qinghai Normal University, Xining, China; ^4^College of Agriculture and Animal Husbandry, Qinghai University, Xining, China

**Keywords:** *Deschampsia caespitosa*, ascorbate-glutathione cycle, stress durations, antioxidant substance and enzyme, water-logging control, drought

## Abstract

Plant growth and development are closely related to water availability. Water deficit and water excess are detrimental to plants, causing a series of damage to plant morphology, physiological and biochemical processes. In the long evolutionary process, plants have evolved an array of complex mechanisms to combat against stressful conditions. In the present study, the duration-dependent changes in ascorbate (AsA) and glutathione (GSH) contents and activities of enzymes involved in the AsA-GSH cycle in hairgrass (*Deschampsia caespitosa*) in response to water stress was investigated in a pot trial using a complete random block design. The treatments were as follows: (1) heavily waterlogging, (2) moderate waterlogging, (3) light waterlogging, (4) light drought, (5) moderate drought, (6) heavily drought, and (7) a control (CK) with plant be maintained at optimum water availability. The hairgrass plants were subjected to waterlogging or drought for 7, 14, 21 and 28 days and data were measured following treatment. Results revealed that hairgrass subjected to water stress can stimulate enzymatic activities of ascorbate peroxidase (APX), glutathione peroxidase (GPX), glutathione reductase (GR), dehydroascorbate reductase (DHAR), monodehydroascorbate reductase (MDHAR) and L-galactono-1, 4-lactone dehydrogenase (GalLDH), switched on the ascorbate-glutathione (AsA-GSH) cycle and the L-galactose synthesis, up-regulated the contents of AsA and GSH, and maintained higher ratios of ascorbate to dehydroascorbate (AsA/DHA) and reduced glutathione to oxidized glutathione (GSH/GSSG) to alleviate potential oxidative damage. However, the light waterlogging did not induce hairgrass under stress to switch on the AsA-GSH pathway. In general, the critic substances and enzyme activities in AsA-GSH metabolic pathway increased as the increase of water stress intensity. As the increase of exposure duration, the critic antioxidant substances content and enzyme activities increased first and then maintained a relatively stable higher level. Our findings provide comprehensive information on biochemical responses of hairgrass to hydrological change, which would be a major step for accelerating ecological restoration of degradation alpine marshes in the Qinghai-Tibetan Plateau.

## Introduction

Water, one of the most critical abiotic factors limiting plant growth, participates in plant physiological and metabolic processes, and affects the survival, growth and distribution of plants. However, the demand of plants to water has a certain threshold. Water excess or water deficit are expected to exert negative effects on plant growth, development, and even plant production, because they may destroy the balance of water (Zupin et al., 2017) and that of reactive oxygen metabolism in plants (Fan et al., 2017; Zupin et al., 2017; Shan et al., 2020). During the long-term evolution, plants have evolved a series of complicated mechanisms, including morphological, physiological, biochemical changes to combat against imbalance of water metabolism and oxidative damage induced by water stress. For instance, plants display remarkably diverse leaf shapes and sizes in nature (Yu et al., 2020; Shi et al., 2021, 2022; Li et al., 2022), and there seems to be a tradeoff between the leaf support cost and photosynthetic returns (Ma et al., 2022; Peng et al., 2022). In addition, when exposed to water deficit, such as in arid and semiarid environment with poor resources, plants are usually with a lower leaf area (Yu et al., 2019; Huang et al., 2021) because water losses and carbon gain processes are tightly coupled (Guo et al., 2021). Although the adoption mechanisms of model plants such as *Arabidopsis thaliana* and important food crops such as *Oryza sativa* (e.g., Damanik et al., 2010) and *Triticum aestivum* (e.g., Lou et al., 2018) to water stress have been intensively studied, our understanding toward that of grass remains limited. Furthermore, numerous studies have revealed that the responses of plants to stress vary depending on plant species or even cultivars, plant development stage and metabolic state, as well as stress intensity and duration (Schneider et al., 2018; Anee et al., 2019; Nemeskéri et al., 2019; Nemeskéri and Helyes, 2019). Therefore, a depth understanding of the response and underlying mechanisms of a target species to water stress is still of paramount importance. The ascorbate-glutathione (AsA-GSH) cycle are found to be crucial in fighting against oxidative damage and keeping the redox balance of plants under water (Shan et al., 2020) or drought stress (Shan et al., 2018), whereas limited literatures have deciphered the responses of plants to alternation of drying and watering yet.

The Qinghai-Tibetan Plateau is the largest and highest highland in the world. In recent years, the Qinghai-Tibetan Plateau had been undergoing significant temperature changes (Yang et al., 2014), which have reshaped the local environment (Yang et al., 2014) and affected local atmospheric circulation and water cycles (Li et al., 2017). Under the background of global warming, drought event frequency, intensity and duration are also expected to increase in the near future due to the combined effects of the decrease in regional precipitation and the increase in evapotranspiration (Trenberth et al., 2013). Meanwhile, precipitation and soil water contents in permafrost region of the Qinghai-Tibetan Plateau are reported to have increased significantly during 1980–2018 (Zhao et al., 2019). In addition, extreme precipitation amount, intensity, frequency and duration have increased evidently (Feng et al., 2020; Guo, 2021). Therefore, the plants in Qinghai-Tibetan Plateau are likely to be subjected to drought and waterlogging stress concurrently. Due to the high altitude and unique alpine climate condition, such as low temperatures, strong evapotranspiration and large direct radiation, the alpine ecosystem is highly fragile and has been suffering from severe degradation due to climate change and anthropic activities during the last decades. The alpine grassland comprises 60% of the total area of the Qinghai-Tibetan Plateau, while about 70% of alpine grassland has been degraded in recent decades (Peng et al., 2019) due to combined effects of climate change and human disturbances (Wang et al., 2015; Zhan et al., 2020; Xu et al., 2022a). The deterioration of grassland greatly restricts the development of local economy and the improvement in living standard of herdsmen, and threats the stability and prosperity of regional society. Besides, grassland degradation undermines its capacity to support biodiversity, ecosystem services and human well-being (Bardgett et al., 2021). To reverse ecosystem deterioration, China has taken various ecological restoration practices, such as grassland cultivation and fencing since 2004. Meanwhile, combating degradation and promoting restoration of grasslands have become one of the most issues for ecological science and policy-making in China (Wang et al., 2015).

An earlier study shows that sowing grass seeds is a feasible and cost-effective way of degraded grassland restoration (Dong et al., 2020). Meanwhile, another study reports that water stress decreases biomass production of alpine grassland plants in autumn (Wang et al., 2020). Therefore, to develop a grass species with strong resilience to water stress maybe a promising alternative. Hairgrass (*Deschampsia caespitosa*) is widely distributed in various habitats, ranging from arid to wet habitats. It has shown highly seed production and germination rate in fields, and thus it is one of potential species used for vegetation restoration in degraded alpine grasslands. However, the physiological responses and underlying mechanisms to water stress remains elusive to date. The major objective of the present study is to investigate the biochemical responses of hairgrass to varying magnitudes and types of water stresses, including different intensities and durations of drought and waterlogging stresses. We specifically addressed the following questions: (1) will drought and waterlogging induced antioxidant response in hairgrass, (2) whether impacts of water stress on hairgrass varied greatly depending on stress duration. By answering these questions, we want to provide a scientific bias and some recommendation for grassland cultivation.

## Materials and methods

### Plant materials

The plant used in the present study is a hairgrass, which is a newly established strain after years of cultivation and domestication in the wild fields. Its seeds were provided by the Grassland Institute, Academy of Veterinary Sciences, Qinghai University. The seeds with highly seed vigor and germination rate and no disease symptom were selected. Selected seeds were surface-sterilized by immersion in a 2% aqueous solution of sodium hypochlorite for 10 min, and five rinses (each time for 5 min) with sterile deionized water.

### Experimental design and growth conditions

The experimental site is located at the Chengbei Campus of the Qinghai Normal University, Xining, Qinghai, China (36°44′31.2′′ N，101°44′56.4′° E), at an altitude of 2390.6 m above mean sea level. The annual mean temperature of this region is 16.4°C. In September 2018, 20 homogenous seeds were sown in each pot (20 cm in diameter, 25 cm in height), which containing 3.0 kg of mixture of alpine meadow soil and sand (sand/soil at 1: 1 volume/volume). The soil was collected from alpine meadow in Dawu Town, Maqing County, Golog Tibetan Ethnic Minority Autonomous Prefecture, Qinghai Province. The alpine meadow soil was classified as Mat Cry-gelic Cambisols (according to the standard of Chinese soil taxonomy research group, 1995). Its chemical characteristics are as follows: total nitrogen 3.12 mg/g, total phosphorus 0.26 mg/g, total potassium 19.58 mg/g, soil organic matter 14.53 mg/g, pH 7.63 (water/soil at1: 1 volume/weight) and CEC 225.52 μS/cm (water/soil at 5: 1 volume/weight). Seeds emerged within 3–5 days later. Seedlings were kept at 10 plants each pot. The plants were kept in the greenhouse and normally watered since then, and were transferred to outdoor during mid-to-late April 2019. Water stress was carried out on July 25 when the grass grew to 25 cm. We applied a complete randomized block experiment design consisting of heavily waterlogging (HW), moderate waterlogging (MW), light waterlogging (LW), control check (CK), light drought (LD), moderate drought (MD) and heavily drought (HD) (Table 1). Each treatment was replicated for 10 times. For plants under drought and normal water demand, we put a basin holder on the bottom of each. For the plants under waterlogging, we place a bucket on the bottom of each basin to prevent water from flowing out. The CK was set as 70–80% of field water-holding capacity, which was measured by ring knife method. Representative undisturbed basin soil was collected with ring knife to absorb water to saturate soil moisture. Gravity water was removed and then dried and weighted. During the study, an awning is built *in situ* and ventilated on both sides of the awning without affecting temperature and humidity. Real-time monitoring of temperature and soil moisture was conducted using portable weather instrument (Hold-HED-SQ, China) and soil moisture sensor (ProCheck, United States), respectively. The water loss was estimated based on daily measurements of pot weight and was supplemented every 2 days. A pot soil without plants was set as a control, and the water loss of soil surface due to evaporation was estimated. Watering was conducted during 18: 00–19: 00. The study lasted for 28 days, and the leaves were collected at 7, 14, 21 and 28 days, respectively, since treatment. Two plants were randomly pruned from 10 pots in the first four times of sampling, and one plant was randomly pruned from 10 pots in the last two times of sampling. After rinsing with distilled water during sampling, the water on leaf surface was wiped. Then, and the leaves of each sample were put into an individual cryopreservation tube and was quickly frozen in liquid nitrogen, and all cryopreservation tubes stored in a refrigerator at-80°C before further proceeding.

**Table 1:** Hydrological conditions used to examine the response of hairgrass (*Deschampsia caespitosa*).

Treatment	Method	Soil water content (%)
Heavily waterlogging, HW	The plants were completely immersed in water	–
Moderate waterlogging, MW	Less than 3 cm of aboveground plants were immersed in water	–
Light waterlogging, LW	The plants were kept at 100% of field water-holding capacity	40%
Control, CK	The plants were kept at 70–80% of field water-holding capacity	28–32%
Light drought, LD	The plants were kept at 50–60% of field water-holding capacity	20–24%
Moderate drought, MD	The plants were kept at 30–40% of field water-holding capacity	12–16%
Heavily drought, HD	The plants were kept at 20% of field water-holding capacity	7–9%

### Determination of the contents of AsA, DHA, GSH and GSSG

The contents of ascorbate (AsA), dehydroascorbate (DHA), reduced glutathione (GSH) and oxidized glutathione (GSSG) were measured as described previously (Hissin and Hilf, 1976; Takahama and Oniki, 1992; Turcsányi et al., 2000). Briefly, for determination of AsA and DHA, 0.5 g of tissue was homogenized on ice in 0.5 ml of 5% metaphosphoric acid and 6 ml of trichloroacetic acid. After centrifugation at 12000 × g for 20 min at 4°C, the supernatants were collected for substrate content determination. For AsA detection, 100 μl of clear homogenate was added with 2 ml of reaction mixture containing 100 mM KH_2_PO_4_ buffer (pH = 6.8) and one unite of ascorbic acid oxidase. The change in absorbance was estimated spectrophotometrically at 265 nm. For determination of DHA, 100 μl of clear homogenate was incubated with added with 2 ml of reaction mixture containing 100 mm KH_2_PO_4_ buffer (pH = 6.8) and 5 μl of 2 mM dithiothreitol (DTT; Sigma-Aldrich, United States), and changes in absorbance was estimated spectrophotometrically at 265 nm. The sum of AsA and DHA and the ratio of AsA/DHA were calculated.

For determination of GSH and GSSG, 0.5 g frozen tissue was ground with sand in mixture of 0.1 mol/l KH_2_PO_4_ buffer (pH = 8.0) containing 5 mm EDTA and 25% phosphoric acid with a mortar and pestle on ice. After centrifugation at 20000 × g for 30 min at 4°C, the supernatants were collected for substrate content determination. For GSH assay, 0.5 ml of original supernatant was mixed with 4.5 ml 0.1 M KH_2_PO_4_ buffer (pH = 8.0) containing 5 mm EDTA and 250 μl 0.1% o-phthalaldehyde (OPA). The mixture was mixed thorough and incubated at room temperature for 15 min. Then, 2 ml final assay solution was transferred to a quartz cuvette. The fluorescence at 420 nm was determined with the activation at 350 nm. For GSSG, 0.5 ml of original supernatant was incubated with 200 μl 0.04 M N-ethylmaleimide (NEM) at room temperature for 30 min. Then, 0.1 M NaOH was added into the mixture. Finally, a 100 μl of the resulted mixture was taken for GSSG measurement using the same procedure of GSH assay. The sum of GSH and GSSG and the ratio of GSH/GSSG were calculated.

### Antioxidant enzyme activity assay

Ascorbate peroxidase (APX) activity was assayed according to standard protocols as described elsewhere (Nakano and Asada, 1981). Briefly, 0.1 g of tissue was homogenized on ice in 3 ml mixture containing 50 mm KH_2_PO_4_ buffer (pH = 7.0), 0.2 mm ethylenediaminetetraacetic acid (EDTA), 1% polyvinylpyrroli done-4,000, 1% Triton X-100 and 5 mm AsA for 12 min. The resulted samples were centrifuged at 13000 × g for 15 min at 4°C, and the supernatants were collected for APX activity assays. The reaction mixture (3 ml) contains 2.6 ml 50 mm KH_2_PO_4_ buffer (including 0.1 Mm EDTA and 0.5 mm AsA), 0.1 ml enzyme extract and 0.3 ml 2 mm H_2_O_2_. The APX activity was determined by dynamically monitoring decreases in ascorbate concentration within 3 min at 290 nm as AsA was oxidized. One unit (nkat) of enzyme was defined as the oxidation of 1 μm AsA per min. The activity of dehydroascorbate reductase (DHAR) was assayed following Omar et al. (2013) with some modification. In brief, 0.5 g tissue was homogenized on ice in 3 ml 50 mm Tris–HCl buffer (pH = 7.2) comprising 0.3 M mannitol, 1 mm EDTA, 0.1% bovine serum albumin (BSA), 0.01% L-Cysteine. After centrifugation at 26000 × g for 20 min at 4°C to remove chloroplast and cell debris, the supernatants were collected. Then, 0.1 ml enzyme extract was added into 3 ml KH_2_PO_4_ buffer (pH = 6.3) containing l mm DHA. After blending thoroughly, the reaction was mixed with 0.1 ml 10 mm GSH. DHAR was assayed by dynamically monitoring changes in absorbance within 3 min at 290 nm. The monodehydroascorbate reductase (MDHAR) activity was assayed following (Miyake and Asada, 1992). The enzyme extract was prepared using the same procedure as described for the DHA extract. The reaction mixture consisted of 3 ml Tris–HCl buffer (pH = 7.2) comprising of 1 M AsA and 0.2 mM NADPH I, 0.1 ml enzyme extract and two units of ascorbic acid oxidase. The MDHAR activity was measured by dynamically monitoring decreases in NADPH I concentration for 3 min at 340 nm. The glutathione reductase (GR) activity was assayed according to Crace and Logan (1996). Specifically, 0.5 g of cells was homogenized on ice in 6 ml mixture containing 50 mm KH_2_PO_4_ buffer (pH = 7.5), 0.1 mm EDTA, 0.3% Triton X-100 and 1% polyvinylpyrrolidone-4,000. The resulted samples were centrifuged at 13000 × g for 15 min at 2°C, and the supernatants were collected for APX activity assays. The reaction mixture (3 ml) contains consisted of 3 ml Tris-KOH buffer (pH = 8.0) comprising 0.5 mm EDTA, 0.5 mm MgCl_2_, 10 mm GSSG, 1 mm NADPH II and enzyme extract. The GR activity was determined by dynamically monitoring changes in absorbance within 3 min at 340 nm. One unit (nkat) of enzyme was defined as the reduction of 1 μm NADPH II per min. The glutathione peroxidase (GPX) was assayed following Khatun et al. (2008). Briefly, 0.50 g tissue was homogenized on ice in 5 ml 0.2 M KH_2_PO_4_ buffer (pH = 6.2) comprising 1 mM EDTA and 5% polyvinylpyrrolidone-4,000. After centrifugation at 8000 × g for 10 min at 4°C to remove chloroplast and cell debris, the supernatants were collected and centrifuged at 12000 × g for 5 min at 4°C. Then, enzyme extract was used to enzymatic activity assay. The GPX activity was assayed by at for 412 nm using H_2_O_2_ as a substrate. The L-galactono-1, 4-lactone dehydrogenase (GalLDH) activity was assayed according to the protocol as described by Li et al. (2009) with some modifications. In brief, 0.3 g tissues were homogenized on ice in 2 ml 100 mm KH_2_PO_4_ buffer (pH = 7.4) comprising 0.4 M sucrose, 10% glycerol (v/v), 1 mm EDTA, 0.3% mercaptoethanol (v/v) and 1% polyvinylpyrrolidone-4,000. After centrifugation at 500 × g for 10 min at 4°C to remove chloroplast and cell debris, the supernatants were collected. Then the supernatants were centrifuged at 12000 × g for 20 min at 4°C. The pellet was suspended in 2 ml 100 mm phosphate buffer (pH = 7.4) containing 5 mm glutathione, 1 mM EDTA and 10% (v/v) glycerol, and the supernatant used for assaying of GalLDH activity. GalLDH activity was assayed following the reduction of cytochrome c at 550 nm at 27°C. The reaction mixture (1 ml) containing 50 mM KH_2_PO_4_ buffer (pH = 7.8), 1.05 mg/ml cytochrome c, 5.6 mm L-galactono-1, 4-lactone and 0.1 ml enzyme extract. Reduction of cytochrome c was started up by adding L-galactono-1, 4-lactone and immediately monitored by the increase in absorbance at 550 nm. One unit (nkat) of enzyme was defined as the oxidation of 1 nm L-galactono-1, 4-lactone per min or the reduction of 2 nm cytochrome c per second.

### Statistical analyses

Statistical analyses were performed with the software package SPSS 22.0 (IBM, Armonk, New York, United States). The data were presented as the mean ± standard error (SE). The assumptions of ANOVA were checked before analysis. Where possible, the effects of hydrological condition, treatment duration and their interactions on parameters were examined with linear mixed models. Differences between hydrological conditions and treatment duration were examined according to the Fisher’s Least Significant Difference (LSD) tests, the significances (*p* ≤ 0.05) were labeled with different uppercase letters or lowercase letters, respectively, in all figures. The figures were produced using OriginPro 2017 (OriginLab Corp, Northampton, United States). The correlations across parameters were examined with Spearman in R version 4.1.3, unless stated otherwise.

## Results

### Dynamic changes in non-enzymatic antioxidant contents

**Figure 1:**
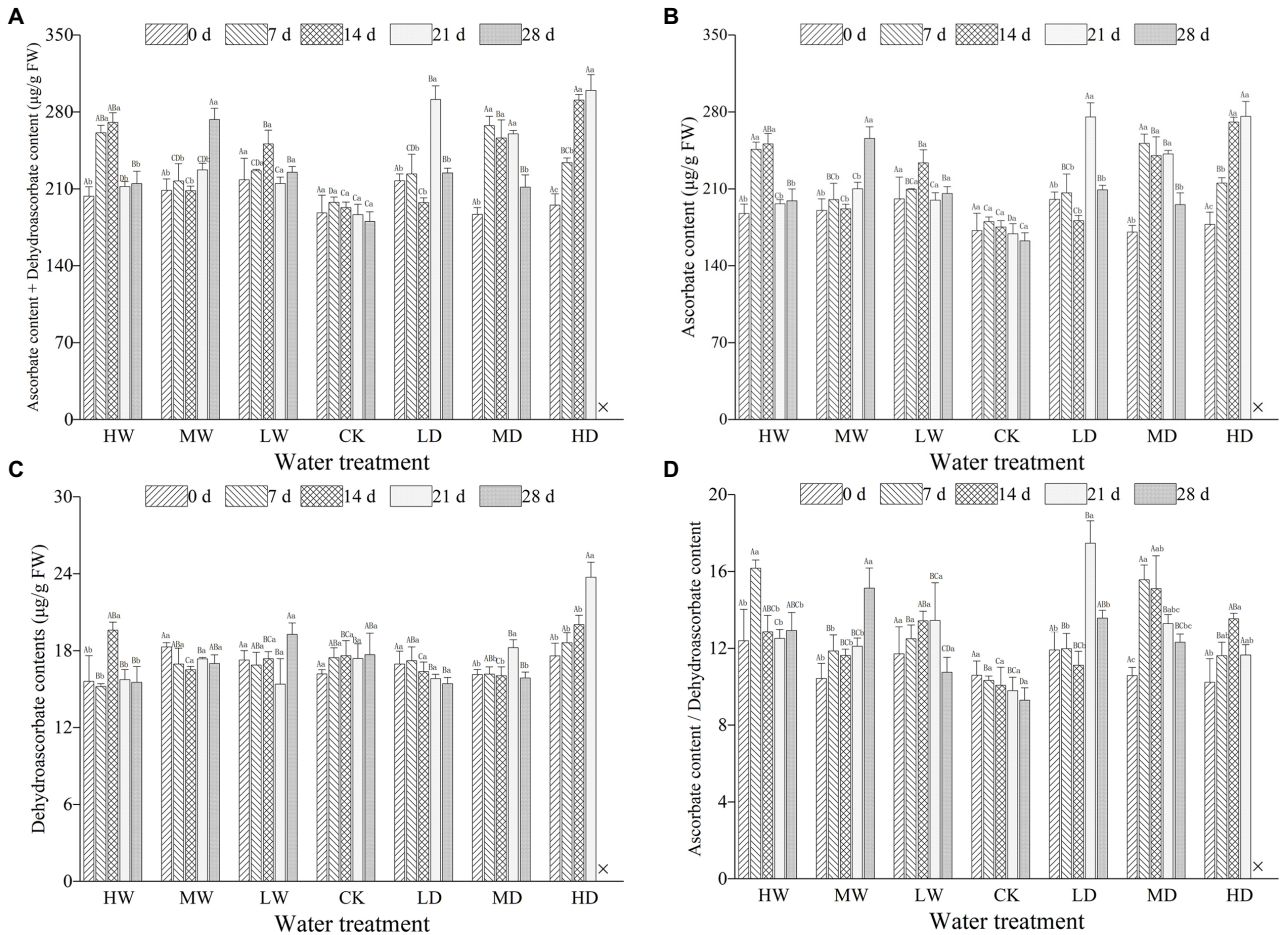
Total ascorbic acid content **(A)**, ascorbate content **(B)**, dehydroascorbate content **(C)** and ratio of ascorbate to dehydroascorbate **(D)** in leaves of hairgrass (*Deschampsia caespitosa*) under contrasting hydrological regimes and durations. Bar values are the mean ± standard error (*N* = 3). Different lowercase letters indicate that there are significant differences among stress exposure durations under the same hydrological treatment; and different capital letters indicate that there are significant differences across different water hydrological regimes at the same exposure duration. X represents the plant died, and thus not allows this parameter to be determined unless stated otherwise. Water treatment are as follows: HW, Heavily waterlogging; MW, Moderate waterlogging; LW, Light waterlogging; CK, Control; LW, Light drought; MW, Moderate drought; and HW, Heavily drought.

**Figure 2:**
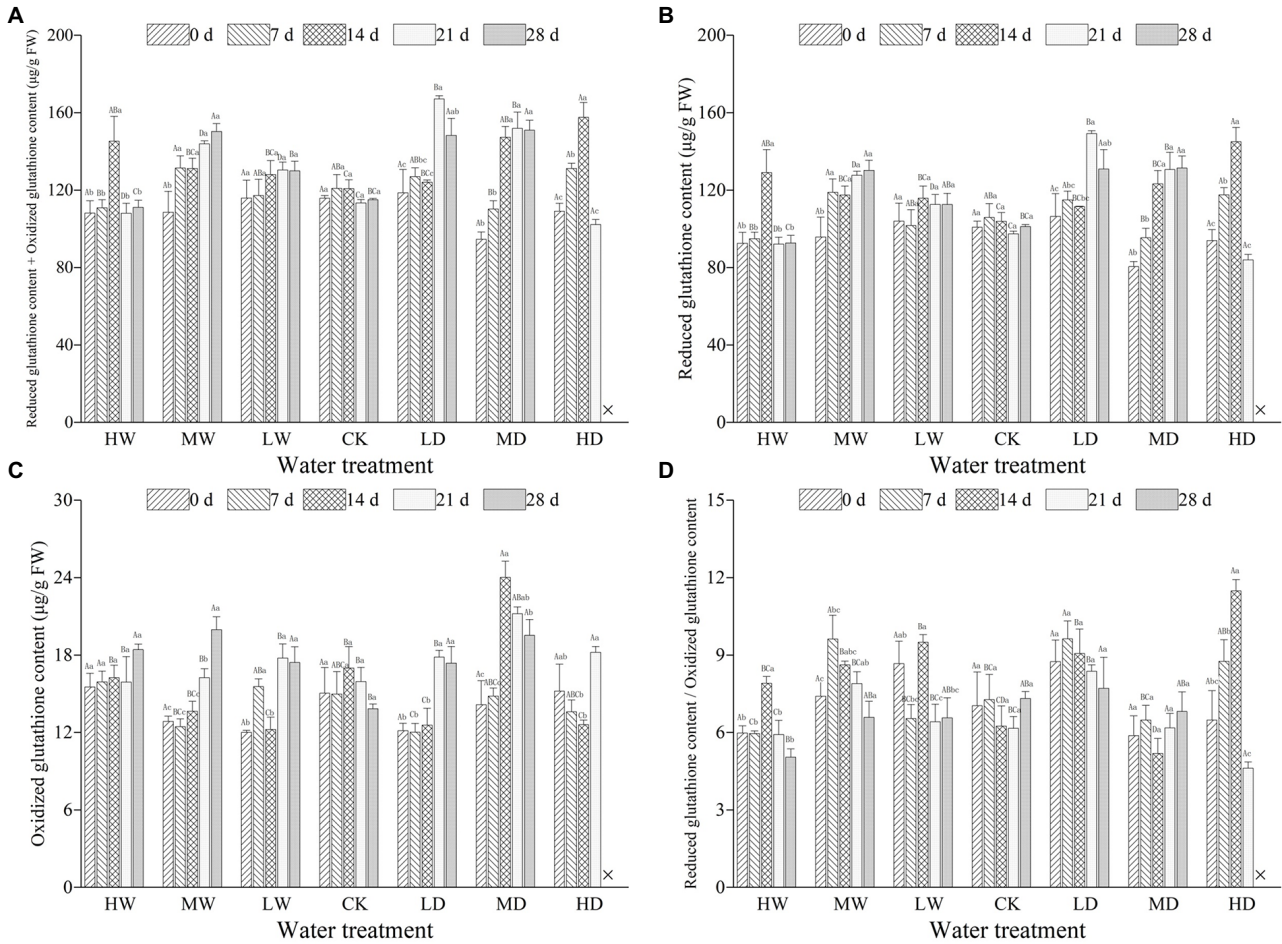
Total glutathione contents **(A)**, reduced glutathione **(B)**, oxidized glutathione **(C)** and ratio of reduced glutathione to oxidized glutathione **(D)** in leaves of hairgrass (*Deschampsia caespitosa*) under contrasting hydrological regimes and durations. Bar values are the mean ± standard error (*N* = 3). Different lowercase letters indicate that there are significant differences among stress exposure durations under the same hydrological treatment; and different capital letters indicate that there are significant differences across different water hydrological regimes at the same exposure duration. X represents the plant died, and thus not allows this parameter to be determined unless stated otherwise. Water treatment are as follows: HW, Heavily waterlogging; MW, Moderate waterlogging; LW, Light waterlogging; CK, Control; LW, Light drought; MW, Moderate drought; and HW, Heavily drought.

Hydrological regime significantly influenced all the parameters used to characterize ascorbic acids. Treatment duration greatly influenced the concentration of AsA and that of AsA + DHA. Meanwhile, the responses of all examined parameters characterizing ascorbic acids to hydrological regime were time-dependent, as revealed by significant interactive effects (Table 2). Hydrological regime, stress duration and their interactions significantly affected contents of GSH, GSSG and GSH + GSSG as well as GSH/GSSG ratio (Table 3). In general, antioxidant contents increased firstly and maintain a relative higher content during the experiment period (Figures 1, 2). The contents of antioxidants are positively correlated to stress magnitude. Neither light waterlogging nor light drought induced significant increase in antioxidant contents, whereas heavily water stress would trigger up-regulation of ascorbic acids within 7 days.

**Table 2:** Results of linear mixed models examining the effects of hydrological regime, stress exposure duration and their interactions on the contents of ascorbic acid in *Deschampsia caespitosa.*

Source of variation	*df*	AsA		DHA		T-AsA		AsA/DHA	
		F	*P*	F	*P*	F	*P*	F	*P*
Hydrological regime (H)	6	17.734	<0.0001	7.667	<0.0001	18.172	<0.0001	8.988	<0.0001
Stress duration (T)	4	16.711	<0.0001	1.168	0.333	17.151	<0.0001	4.647	0.002
H × T	23	7.875	<0.0001	2.416	0.003	8.093	<0.0001	3.386	<0.0001

AsA, ascorbic acid; DHA, dehydroascorbate; T-AsA, the sum of reduced and oxidized ascorbate; AsA/DHA, the ratio of reduced to oxidized ascorbate.

**Table 3:** Results of linear mixed models examining the effects of hydrological regime, stress duration and their interactions on the glutathione in *Deschampsia caespitosa.*

Source of variation	*df*	GSH		GSSG		T-GSH		GSH/GSSG	
		F	*P*	F	*P*	F	*P*	F	*P*
Hydrological regime (H)	6	8.310	<0.0001	8.525	<0.0001	7.568	<0.001	9.921	<0.0001
Stress duration (T)	4	16.227	<0.0001	17.957	<0.0001	21.323	<0.0001	7.701	<0.0001
H × T	23	5.591	<0.0001	4.090	<0.0001	6.202	<0.0001	4.304	<0.0001

GSH, glutathione; GSSG, glutathione oxidized; T-GSH, the sum of reduced and oxidized glutathione; GSH/GSSG, the ratio of reduced to oxidized glutathione.

### Dynamic changes in antioxidant enzymatic activities

**Figure 3:**
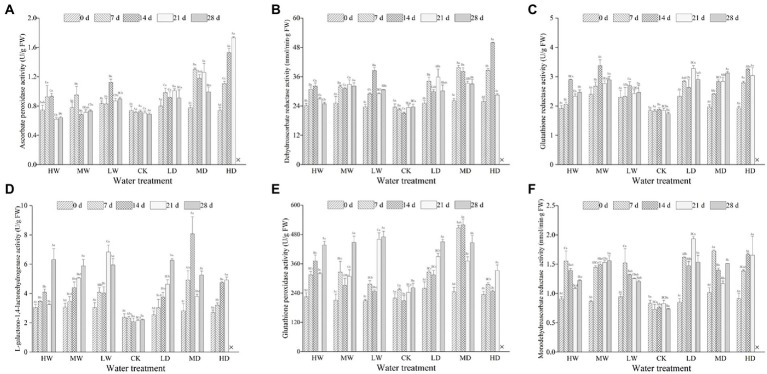
Enzymatic activities of ascorbate peroxidase **(A)**, dehydroascorbate reductase **(B)**, glutathione reductase **(C)**, L-galactono-1, 4-lactone dehydrogenase **(D)**, glutathione peroxidase **(E)** and monodehydroascorbate reductase **(F)** in leaves of hairgrass (*Deschampsia caespitosa*) under different hydrological regimes. Bar values are the mean ± standard error (*N* = 3). Different lowercase letters indicate that there are significant differences among stress exposure durations under the same hydrological treatment; and different capital letters indicate that there are significant differences across different water hydrological regimes at the same exposure duration. X represents the plant died, and thus not allows this parameter to be determined unless stated otherwise. Water treatment are as follows: HW, Heavily waterlogging; MW, Moderate waterlogging; LW, Light waterlogging; CK, Control; LW, Light drought; MW, Moderate drought; and HW, Heavily drought.

Hydrological regime, exposure duration and their interactions significantly affected the activities of APX, DHAR, MDHAR, GalLDH, GR and GPX (Table 4). As shown in Figure 3, antioxidant enzymatic activities are positively correlated to stress magnitude. They increased significantly within 7 days of exposure and maintain a relative higher content during the experiment period. Light water stress did not induce up-regulation of defense enzymatic activity. Meanwhile, the response of antioxidant enzymatic activities to water stress depends on enzymatic identity.

**Table 4:** Results of linear mixed models examining the effects of hydrological regime, stress duration and their interactions on the critical enzyme activities in AsA-GSH cycle of hairgrass (*Deschampsia caespitosa*).

Parameters	Hydrological regime (H)			Stress duration (T)			H × T		
	*df*	*F*	*P*	*df*	*F*	*P*	*df*	*F*	*P*
GalLDH	6	28.177	<0.0001	4	42.725	<0.0001	23	7.795	<0.0001
APX	6	46.745	<0.0001	4	18.445	0.001	23	8.363	<0.0001
DHAR	6	50.319	<0.0001	4	54.202	<0.0001	23	10.335	<0.0001
MDHAR	6	34.970	<0.0001	4	38.882	<0.0001	23	4.849	<0.0001
GR	6	30.041	<0.0001	4	24.275	<0.0001	23	3.085	<0.0001
GPX	6	37.712	<0.0001	4	72.324	<0.0001	23	9.032	<0.0001

GalLDH, L-galactono-1,4-lactone dehydrogenase, APX, Ascorbate peroxidase, DHAR, Dehydroascorbate reductase, MDHAR, Monodehydroascorbate reductase, GR, glutathione reductase, GPX, glutathione peroxidase.

### Correlations between non-enzymatic antioxidant content and antioxidant enzymatic activity

**Figure 4:**
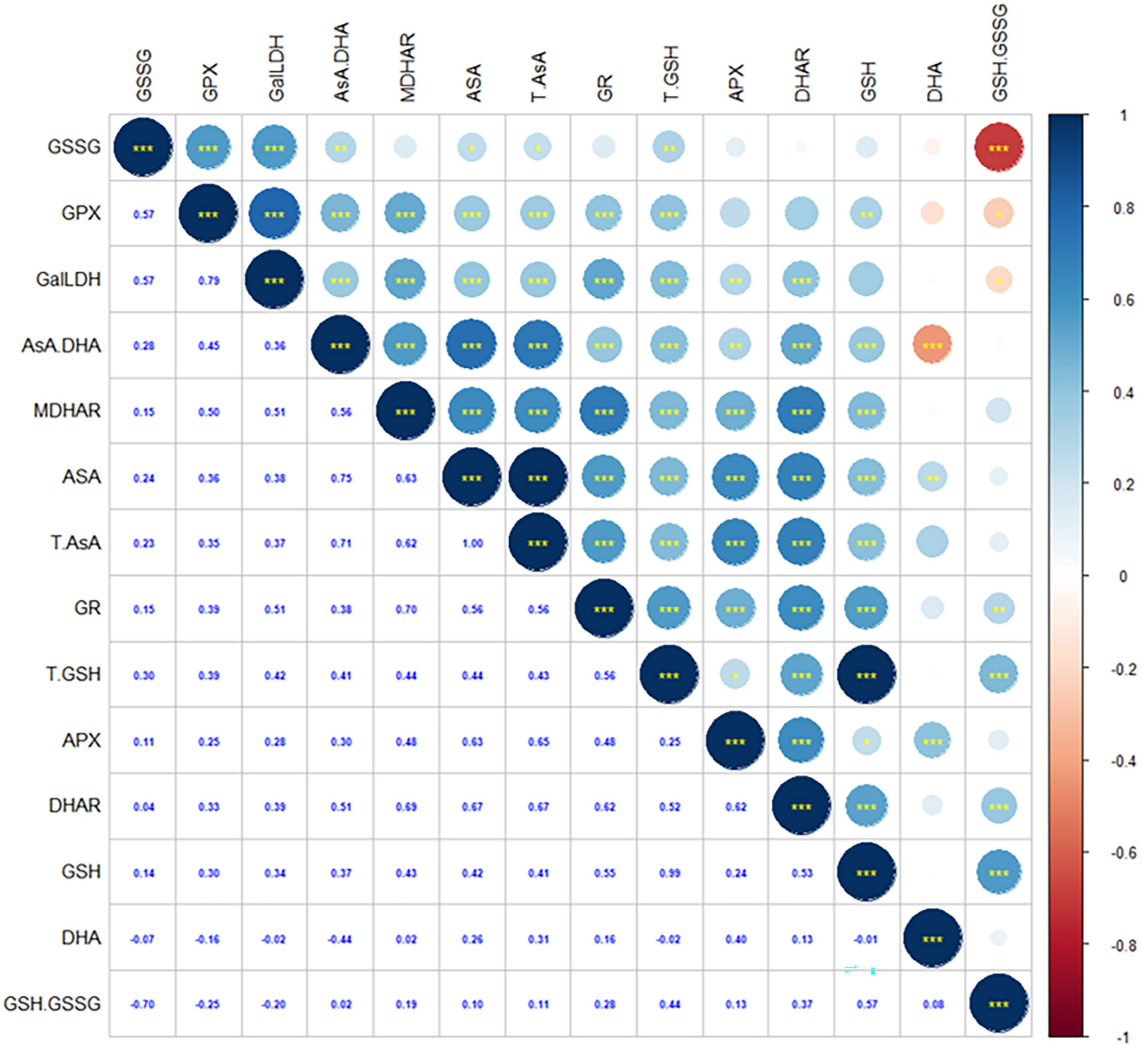
The Spearman’s correlation coefficient between non-enzymatic substance accumlation and enzyme activities in AsA-GSH cycle of hairgrass (*Deschampsia caespitosa*) under different hydrological regimes. AsA---ascorbate, DHA---dehydroascorbate, T-AsA, the sum of ascorbate and dehydroascorbate; AsA/DHA, the ratio of ascorbate to dehydroascorbate; GSH, reduced glutathione; GSSG, oxidized glutathione; T-GSH, the sum of reduced glutathione and oxidized glutathione; GSH/GSSG, the ratio of reduced glutathione to oxidized glutathione; APX, ascorbate peroxidase; DHAR, dehydroascorbate reductase; GPX, glutathione peroxidase; GR, glutathione reductase; GalLDH, L-galactono-1; 4-lactone dehydrogenase; MDHAR, monodehydroascorbate reductase.

As shown in Figure 4, AsA content is positively correlated to DHA content, T-AsA content, AsA/DHA, GSH content, GSSG content, T-GSH content, APX activity, DHAR activity, MDHAR activity, GalLDH activity, GR activity and GPX activity. DHA content is positively correlated to AsA content, T-AsA content and APX activity, whereas negatively correlated to AsA/DHA. AsA/DHA is positively correlated to AsA content, T-AsA content, GSH content, GSSG content, T-GSH content, APX activity, DHAR activity, MDHAR activity, GalLDH activity, GR activity and GPX activity, whereas negatively correlated to DHA content. GSH content is positively correlated to AsA content, T-AsA content, AsA/DHA, T-GSH content, GSH/GSSG, APX activity, DHAR activity, MDHAR activity, GalLDH activity, GR activity and GPX activity. GSSG content is positively correlated to AsA content, T-AsA content, AsA/DHA, T-GSH content, GalLDH activity, DHAR activity, GR activity and GPX activity, whereas negatively correlated to GSH/GSSG. GSH/GSSG is positively correlated to GSH content, T-GSH content, MDHAR activity and GR activity, whereas negatively correlated to GSSG content, GalLDH activity and GPX activity.

## Discussion

Since plant functional traits are correlated to many ecological processes (Cornwell and Cornelissen, 2008; Chin and Potts, 2018), deciphering the complex responses of plant functional traits under various environmental conditions was a better method to evaluate the potential impacts of global change. The current study examined the changes in non-enzymatic antioxidant contents and antioxidant enzyme activities under contrasting water stresses. On the whole, we observed that hairgrass enhanced non-enzymatic antioxidant accumulation and up-regulated antioxidant enzymatic activities in response to water stress. However, hairgrass exposed to light water stress did not innate defense response, indicating a trade-off between tolerance and oxidant damage defense. As reported, one functional trait would be strengthen at the expense of the construction and functional maintenance of other traits when plant resources are limited (e.g., Yu et al., 2019).

### Contents of AsA, DHA, GSSG and GSH

AsA, an important water soluble non-enzymatic antioxidant with a low molecular weight, plays crucial roles in plant growth, metabolisms, development and stress response (Li et al., 2009; Anjum et al., 2014; Mostofa et al., 2015). At present, the contents of AsA are regulated by four synthesis pathways and one regeneration pathway. Specifically, the L-galactose pathway is recognized as the most important pathway for higher plants to synthesize AsA, the D-galacturonic acid pathway the auxiliary pathway, L-glucose pathway and inositol pathway the supplementary pathway, and AsA-GSH cycle pathway the regeneration pathway. The AsA/DHA ratio is an indicator of stress intensity experienced by plants (Kampfenkel et al., 1995), and changes in the AsA/DHA ratio are considered as an indicator of redox status in plants (Foyer and Noctor, 2011; Anjum et al., 2014). In the present study, the AsA and DHA contents of hairgrass plants under heavily waterlogging was increased in comparison with control after 7 days and 14 days. Our findings are in contrast with Anee et al. (2019), in which AsA and DHA of *Sesamum indicum* under waterlogging was steadily increased after 2, 4, 6, 8 days in comparison with control. In addition, the AsA and DHA contents of hairgrass plants under heavily drought were steadily increased in our study (Figure 1). Earlier studies reported that drought at 30% of field water-holding capacity evidently increased the accumulation non-enzymatic antioxidants (AsA and GSH), which are vital for ROS detoxification in the *Amaranthus tricolor* (Sarker and Oba, 2018). One plausible explanation for the discrepancy would be that the species-specific as well intensity and duration dependent of plants to water stress. Further studies are needed to decipher the short-term response of AsA and DHA to water stress.

GSH is a water-soluble thiol compound of low molecular weight, widely distributed in most plant tissues and participates directly or indirectly in detoxification of ROS (Foyer and Noctor, 2005). Besides, GSH is also an important component of antioxidant regeneration system, involved in recycling of AsA and regulating the redox homeostasis (de Carvalho, 2008; Szarka et al., 2012; Gruszka et al., 2018). Furthermore, GSH, a substrate for GPX, detoxifies lipid hydroperoxides together with GSTs, and the GSH/GSSG induces signals for abiotic stress adaptation (Hasanuzzaman et al., 2012). The accumulation of GSSG is often associated with tissue death or quiescence (Foyer and Noctor, 2005). In the present study, the changes in GSH and GSSG contents of plants did not show a regular change in response to waterlogging (Figure 2). Our findings are in contrast to the reported increased GSH and GSSG contents for plants whereas did not have affect GSH/GSSG under waterlogging (Anee et al., 2019). However, drought tend to increase GSH and GSSG contents (Figure 2). This result is in line with previous studies (e.g., Nahar et al., 2015; Gruszka et al., 2018; Lou et al., 2018). Drought exposure duration at 35–40% of field water-holding capacity is reported to result in reduced GSH/GSSG in the *Triticum aestivum* (Lou et al., 2018). Furthermore, drought increased the accumulation non-enzymatic antioxidants (AsA and GSH) of barley (Gruszka et al., 2018), but declined the ratios of AsA/DHA and GSH/GSSG in leaves of wheat seedling (Shan et al., 2018).

Collectively, we observed increased contents of AsA, DHA, AsA + DHA, GSH, GSSG and GSH + GSSG whereas reduced of AsA/DHA and GSH/GSSG of the hairgrass under water stress in the present study, indicating water stress can induce hairgrass to up-regulate non-enzymatic antioxidant contents to alleviate the negative impacts of water stress on plant growth (Gruszka et al., 2018; El-Beltagi et al., 2020).

### Antioxidant enzyme activities

Previous studies have suggested that antioxidant enzymes of the AsA-GSH cycle, comprising the APX, MDHAR, DHAR and GR, play a vital role in detoxifying reactive oxygen species (Asada, 1999; Mostofa et al., 2015; Hasanuzzaman et al., 2019). APXs are heme-binding enzymes that reduce hydrogen peroxide to water utilizing AsA as an electron donor (Asada, 1999), participating in the AsA-GSH cycle or Foyer–Halliwell–Asada pathway (Anjum et al., 2016; Mukarram et al., 2021). Over-expression of APX has been shown to increase drought tolerance (Faize et al., 2011; Xu et al., 2022b). As a critical enzyme involved in AsA recycling (Liu et al., 2022), the DHAR reduces DHA to AsA using GSH as substrate. In addition, the MDHAR reduces MDHA to AsA, and the GR reduces the GSSG back to GSH (Anjum et al., 2014, 2016). GalLDH is a key enzyme in the last step of AsA biosynthesis in the Smifnoff-Wheeler pathway (Anjum et al., 2014).

Empirical studies have confirmed that up-regulation or over-expression of AsA-GSH pathway enzymes and the enhancement of the AsA and GSH levels conferred plants better tolerance to abiotic stresses by reducing the ROS (Anee et al., 2019; Hasanuzzaman et al., 2019; Wang et al., 2022). Drought up-regulated the enzymatic antioxidant activities of AsA-GSH pool (Hasanuzzaman et al., 2017; Bhuiyan et al., 2019), and enzymatic response of AsA-GSH pathways varied depending upon plant species (Gruszka et al., 2018) and plant developmental stage, as well as drought intensity and duration (Hasanuzzaman et al., 2017). Similarly, enzymatic response of AsA-GSH pathways to waterlogging stress also varied depending upon the plant species (Damanik et al., 2010; Sairam et al., 2010; Simova-Stoilova et al., 2012; Wang et al., 2019) and stress duration (Anee et al., 2019; Hasanuzzaman et al., 2019). In a Meta-analysis, water stress did not affect the activity of APX and GR (Sun et al., 2020). Anee et al. (2019) reported the APX and MDHAR activity increased with stress duration while DHAR and GR activity reduced when plants subjected to waterlogging. Xu et al. (2022b) found enhanced activities of APX and GR in response to drought. In our study a time-dependent pattern of antioxidant enzyme activities to water stress was observed (Figure 3).

### Correlations between non-enzymatic antioxidant contents and antioxidant enzyme activities

As reported, AsA can be biosynthesized through L-galactose pathway (Li et al., 2009; Liao et al., 2021). MDHAR is critic for AsA regeneration; MDHAR and DHAR maintain the DHA pool and control the AsA/DHA (Anjum et al., 2014). Previous studies have demonstrated a positive correlation between the activities of MDHAR and DHAR, and between the contents of T-AsA and the AsA/DHA ratio in kiwifruit (Liao et al., 2021). AsA content is positively correlated APX activity, DHAR activity, MDHAR activity, GalLDH activity, GR activity and GPX activity (Figure 4). This is a logical conclusion. Similarly, GR is responsible for the regeneration of GSH (Shan et al., 2018), and thus maintains the GSH pool. Higher GPX activity may impose a threat to the level of GSH and the GSH/GGSG ratio in the absence of efficient recycling of GSSG (Mostofa et al., 2015). Taken together, our correlation analyses displayed intricate links between non-enzymatic antioxidants and antioxidant enzymes in protecting plants from potential damage posed by water stress.

## Conclusion

In summary, the biochemical responses of hairgrass to hydrological changes depend on stress intensity and duration. Specifically, water stress induced increases in contents of AsA, DHA, AsA + DHA, GSH, GSSG and GSH + GSSG but reduced the ratios of AsA/DHA and GSH/GSSG. In addition, it enhanced the activities of APX, DHAR, GPX, GR, MDHAR and GalLDH, and switched on the AsA-GSH cycle pathway and the L-galactose synthesis pathway to mitigate potential oxidative damage posed by water stress. The light waterlogging did not induce hairgrass to initiate defense response, indicating that hairgrass has developed resistance against light waterlogging stress. Finally, the response of hairgrass to water stress (including waterlogging and drought) decreased with experimental duration; implying hairgrass has a great capacity to adapt to water stress.

## Data availability statement

The original contributions presented in the study are included in the article/Supplementary material; further inquiries can be directed to the corresponding author.

## Author contributions

YsM designed the experiments. QL and YgM collected the samples. QL, YW, and LZ performed the laboratory work. QL, HX, and ZC analyzed the data. QL wrote the first version of the manuscript, which was then edited by all co-authors. All authors contributed to the article and approved the submitted version.

## Funding

This work was kindly supported by the Second Comprehensive Scientific Investigation and Research Project of Qinghai-Tibet Plateau (SQ2019QZKK2206), the National Key Research and Development Program of China (2016YFC0501903), Natural Science Foundation of Qinghai Province (2022-ZJ-941Q); University-level Young and Middle-aged Scientific Research Fund Project (KJQN2021003).

## Conflict of interest

The authors declare that the research was conducted in the absence of any commercial or financial relationships that could be construed as a potential conflict of interest.

## Publisher’s note

All claims expressed in this article are solely those of the authors and do not necessarily represent those of their affiliated organizations, or those of the publisher, the editors and the reviewers. Any product that may be evaluated in this article, or claim that may be made by its manufacturer, is not guaranteed or endorsed by the publisher.
